# Rational therapeutic targeting of myeloid cells in glioblastoma: challenges and perspectives

**DOI:** 10.3389/fimmu.2025.1472710

**Published:** 2025-06-26

**Authors:** Faruk Akay, Maya Saleh

**Affiliations:** Institut National de la Recherche Scientifique (INRS), Centre Armand-Frappier Sante Biotechnologie, Laval, QC, Canada

**Keywords:** central nervous system, glioblastoma, tumor microenvironment, innate immunity, myeloid cells, glioma-associated macrophages, immunotherapy, clinical trials

## Abstract

Glioblastoma (GB) is the most aggressive tumor of the central nervous system (CNS), accounting for almost 80% of all primary brain tumors. Despite standard-of-care consisting of surgical resection, when possible, adjuvant radiotherapy (RT) and chemotherapy with Temozolomide (TMZ), GB remains highly fatal, with an estimated recurrence rate of over 90% and a median overall survival (OS) of around 15 months from diagnosis. Several factors contribute to such poor patient outcome, including a unique myeloid-rich tumor microenvironment (TME) that confers immunosuppression and therapeutic resistance. Multi-omics, single-cell transcriptomics and multi-modal spatial analyses of GB are unraveling the diversity of brain myeloid cells, including activated microglia, border-associated macrophages (BAM), and monocyte-derived glioma-associated macrophages (GAM), instructed by ontogeny, spatial distribution, cell-cell interactions and response to metabolic cues in the TME. In this review, we elaborate on the heterogeneity and plasticity of myeloid cells in GB and discuss the promise and challenges for rational therapeutic targeting of GAMs in GB.

## 1 Glioblastoma classification and current standard-of-care

**Figure 1:**
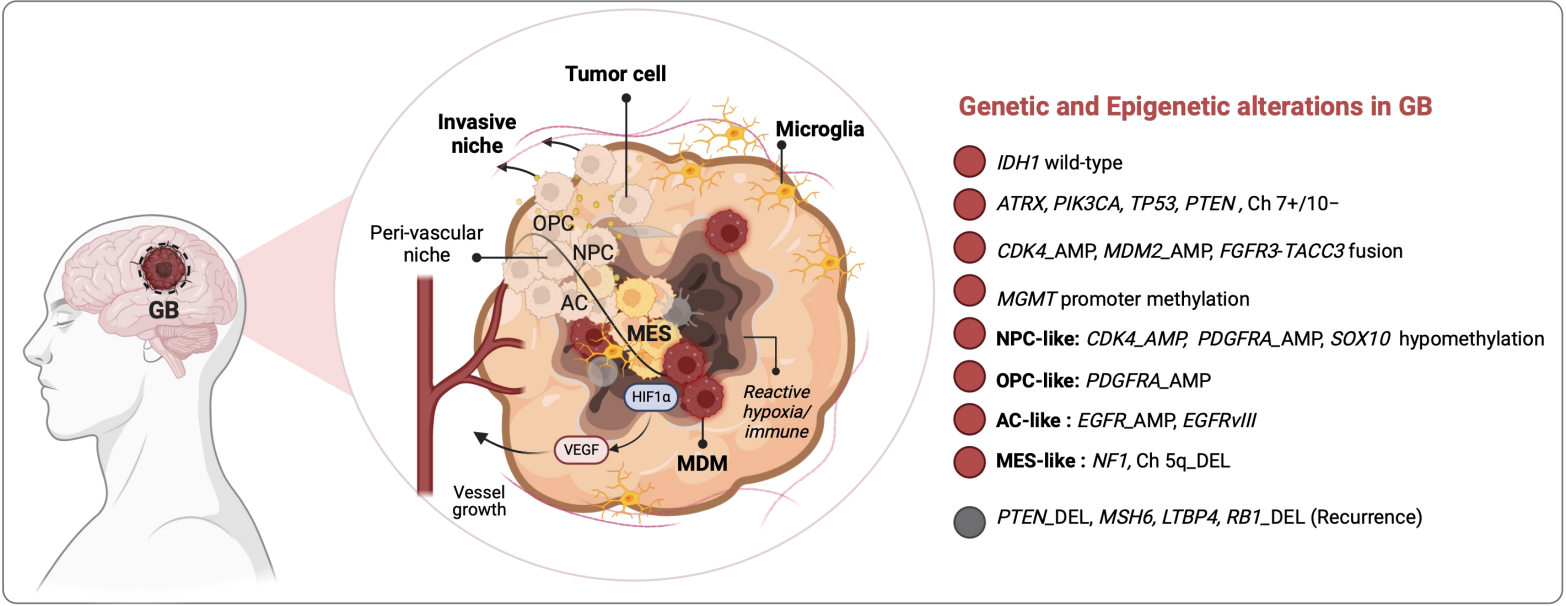
Glioma-TME interactions drive tumoral heterogeneity and therapeutic resistance in patients with GB. Inter-and intra-tumoral heterogeneity (ITH) in GB is governed by genetic and epigenetic drivers, as well as by tumor microenvironment (TME) signals that shape glioma stem cell (GSC) programs into NPC-, OPC-, AC- or MES-like states according to stresses in different tumor niches i.e., peri-vascular, hypoxic and peri-necrotic zones. NPC, neural progenitor cells; OPC, oligodendrocyte progenitor cells; AC, astrocyte cells; MES, mesenchymal. HIF1α, hypoxia inducible factor 1 subunit alpha; VEGF, vascular endothelial growth factor; Ch, chromosome; AMP, amplification; DEL, deletion.

Brain cancer ranks 12^th^ among the deadliest cancers worldwide (1). According to the 5^th^ edition of the WHO classification of CNS tumors, 30% of gliomas are low-grade gliomas (LGG), whereas the rest (70%) are diffuse and infiltrative GB (grade IV). Molecularly, GB are isocitrate dehydrogenase (*IDH)1* wild-type (WT) but harbor genetic and epigenetic alterations mainly in Epidermal Growth Factor Receptor (*EGFR*), Platelet-Derived Growth Factor Receptor α (*PDGFRA*), Cyclin Dependent Kinase Inhibitor 2A (*CDKN2A*), Neurofibromatosis type 1 (NF1), Phosphatase and Tensin Homolog (*PTEN*), Tumor Protein p53 (*TP53*) and the dual gain of chromosome 7 and loss of chromosome 10 (2) (**Figure 1**). In ~50% of the cases, the promoter of the DNA repair gene O-6-methylguanine-DNA methyltransferase (*MGMT*) is hypomethylated, which contributes to chemoresistance. The current standard-of-care for patients with newly diagnosed GB follows the STUPP protocol, consisting of a multimodal approach of maximal safe resection surgery, followed by radiation and adjuvant oral chemotherapy with TMZ (3). To prevent seizures or brain edema, patients with GB are additionally prescribed antiepileptic medications, deep vein thrombosis prophylaxis and steroids (4). However, despite standard-of-care, the OS of patients with GB is estimated at 0.71% (5), and such bleak outcome is thought to stem from rapid regrowth of invasive cells post-treatment.

## 2 GB plasticity governed by TME metabolic and immune signals

GB progression is driven by genetic evolution and glioma stem cell (GSC) plasticity in response to TME signals (6). Through single-cell RNA sequencing (scRNA-seq), and analysis of bulk transcriptomic data of GB tumors from the Cancer Genome Atlas (TCGA), Neftel et al. identified 4 GB cellular states: The first three share features with cell lineages of the normal fetal brain and are accordingly referred as neural progenitor-like (NPC-like), oligodendrocyte-progenitor-like (OPC-like), astrocyte-like (AC-like), whereas the fourth, referred to as the mesenchymal-like (MES-like) subtype, does not have a direct parallel with normal progenitors. These states are driven by distinct genetic aberrations, e.g., in *EGFR*, *PDGFRA*, and *NF1* that lead to NPC-like, AC-like and MES-like, respectively (**Figure 1**). On the other hand, deletion of chromosome arm 5q negatively correlated with the MES-like state, presumably due to loss of several MES state regulators encoded by this region, such as Mothers against decapentaplegic homolog 5 (also known as *SMAD5*), Transforming growth factor beta 1 (*TGFB1*), Colony stimulating factor 2 (*CSF2*), *IL3/4/13*, and C-X-C motif chemokine ligand 14 (*CXCL14*) (6, 7). Consistent with the results from Neftel et al. a spatial multiomics analysis by Moffet et al. revealed that despite ITH two main tumor cell states emerge across GB patient cohorts: glial-like tumors associated with microglia and MES-like tumors dominated by MDMs (8).

Several factors, including metabolic and immune signals, have been implicated in GSC plasticity. Among these, hypoxia figures as a major tissue organizer (9) impacting the phenotypes as well as dialogue between GSC and myeloid cells. Ravi et al. used spatially resolved muti-omics and showed that the MES-like state segregated with regions of “reactive immune hypoxia”, pointing to a link between environmental stress and GSC adaptations (10). Among the factors driving the MES state are inflammatory signals converging on the activation of the master transcription factors Signal transducer and activator of transcription 3 (STAT3), Activator Protein 1 (AP-1) and Nuclear factor kappa B (NF-κB). Hara et al. demonstrated that the GAM-derived IL-6 family cytokine, oncostatin M (OSM), promoted the MES-like state of GB via OSM receptor (OSMR) and STAT3 signaling in GSC (11). Chen et al. identified FOS-like antigen 1 (FOSL1), a component of AP-1 as an additional factor involved in the in proneural to mesenchymal transition. FOSL1 promoted a MES-like state through UBC9-dependent SUMOylation of Cylindromatosis Y-like loss deubiquitinase (CYLD) and K63-linked polyubiquitination and activation of NF-κB (12). Concordantly, Wang et al. demonstrated a direct role of AP-1 in driving the MES state phenotypic switch in recurrent GB (13). Besides AP-1, post-treatment recurrent GB cells with a MES-like phenotype adapt a “VC-Resist” (vessel co-opting and resistant) state dependent on Fibroblast Growth Factor Receptor 1 (FGFR1), Yes-Associated Protein 1 (YAP1), the HIPPO pathway and senescence induction which allow them to infiltrate in the surrounding brain and “hide” in peri-vascular niches (14).

Besides GSC regulation, hypoxia also sculpts the TME. Notably, a spatial immune profiling of human GB showed distinct immune landscapes in the tumor peri-vascular (PVZ) versus peri-necrotic (PNZ) zones, which correlated with patient survival (15). TME components are increasingly appreciated as important effectors of clinical outcomes. Hoogstrate and colleagues applied bulk transcriptomics on paired primary-recurrent GB tumors from patients on standard-of-care (n = 322 test, n = 245 validation), together with scRNA-seq and immunohistochemistry validation, and showed that GB progression occurred through TME remodeling rather than molecular alterations in GB evolution, which might explain the limited success of targeted therapies in GB (16). Their results revealed recurrence-associated decrease in endothelial cells countered by decreased tumor ‘purity’ with increased neuron/oligodendrocyte/tumor co-occurrence, enhanced GAMs and pericytes and increased extracellular matrix remodeling (16). Among the GAMs, Luo et al. identified matrix metalloproteinase (MMP)14^+^ myeloid cells as critical promoter of glioma angiogenesis and associated with improved OS in response to TMZ in combination with anti-angiogenic bevacizumab (anti-VEGF) treatment (17).Therefore, to refine target discovery, current research is focused on mapping TME cellular diversity at single cell resolution both in patient cohorts and in relevant *in vivo* and *ex vivo* models e.g., using syngeneic orthotopic glioma models (18–20), new reporter mice (e.g., Tmem119^GFP^ and Hexb^CreERT2^ for microglia) (21, 22), human organotypic brain slice culture (23) and advanced microscopy (24, 25).

## 3 The diversity and functions of myeloid cells in GB

### 3.1 Myeloid cell landscape in GB

**Figure 2:**
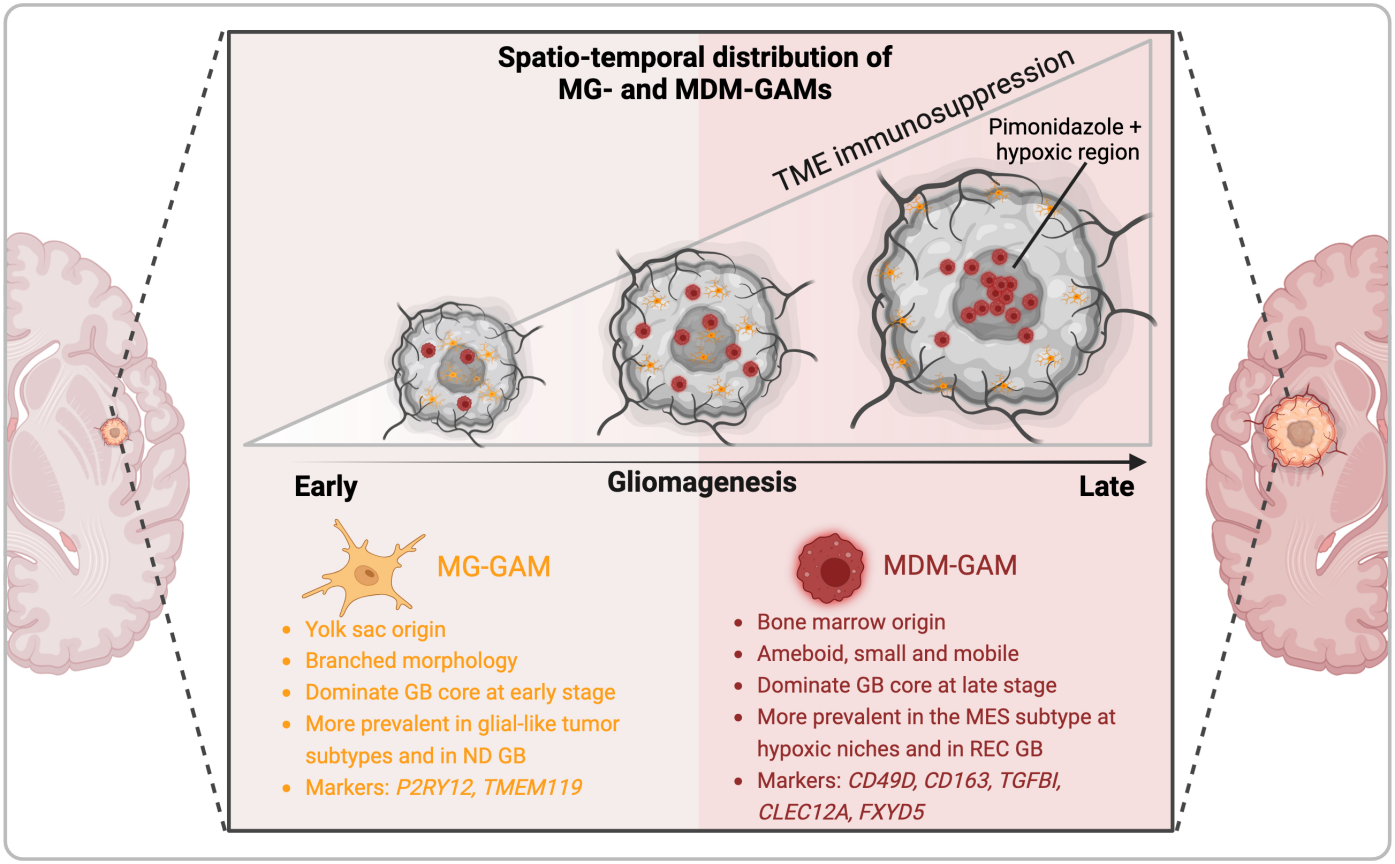
Spatiotemporal distribution and diversity of glioma-associated macrophages in GB. The tumor immune microenvironment of GB is dominated by fetal-derived microglia (MG) and monocyte-derived macrophages (MDM) that form the pool of glioma-associated macrophages (GAMs). Single cell and spatially-resolved omics in mouse models and patients samples together with advanced microscopy analyses revealed that MG-GAM predominate in early gliomagenesis, in glial-like tumors (NPC, OPC, AS) and in newly diagnosed (ND) GB but are excluded from the tumor core by the influx of MDM that predominate in advanced tumors, especially of the MES-like subtype, and in recurrent (REC) GB.

A better understanding of the morphology, diversity and function of GAMs is needed to improve their rationale targeting. Sankowski et al. were among the first to characterize the states of microglia in adult human patients who have undergone brain surgery to resect epileptic foci (n = 10), gliomas (n = 4) or brain metastases (n = 1) (26). They implemented scRNA-seq and time-of-flight mass cytometry (CYTOF) to identify regional and age-associated heterogeneity in microglia phenotypes including a) higher abundance of activated microglia in the white matter compared to the gray matter; b) common features expressed by all subsets of microglia (e.g., *CX3CR1, TMEM119, CSF1R, P2RY12, SELPLG, MARCKS*) versus discriminating features that identify specialized functions e.g. in antigen presentation (*HLA-DRA, CD74*), chemotaxis/inflammation (*CCL2, TNF, IL1B*), or hypoxia-response/angiogenesis (*HIF1A, VEGFA*); and c) downregulation of microglia homeostatic genes countered by upregulation of metabolic (*APOE, LPL*), inflammatory (*SPP1*) and interferon-induced (*IFI27, IFITM3*) genes in aging and in GB tumors (26). Batchu et al. profiled the immune landscapes of *IDH1* WT GB versus *IDH1* mutant astrocytoma (27). They identified 7 GAM subtypes present in both, including interferon-primed (IFN-), immunoregulatory (reg-), cytokine-enriched (inflam-), lipid-associated (LA-), pro-angiogenic (angio-), tissue resident-like (RTM-), and proliferating (prolif-) GAM subsets. Ligand-receptor maps showed that all 7 GAM subpopulations strongly interacted with T cells in *IDH1* WT GB while only two subsets (inflam-GAMs and LA-GAMs) did in *IDH1* mutant tumors. GAM-T cell crosstalk correlated with signatures of T cell exhaustion (*PD-1, LAG-3, TIM-3, CTLA-4, TIGIT*) and of inhibited T cell migration, highlighting different immunological ecosystems in these two advanced glioma types and a more immunosuppressive environment in *IDH1* WT GB. Among the pathways engaged in all 7 GAM subpopulations in GB were those involving SPP1, MIF, GALECTIN, COMPLEMENT and PTN (Pleiotrophin), unraveling these pathways as potential immunosuppressive and pro-tumoral effectors (27). These phenotypes were confirmed in glioma mouse models, particularly a glioma-associated shift to phagocytic and DC-like states. These are evidenced by upregulation of *CD11c*, genes associated with migration (*Cxcl13, Cx3Cr1, Csf1r*), actin cytoskeleton regulation (*Fscn1, Coro1a*), cholesterol homeostasis (*Abca1, Abcg1*) and endocytosis (*Apoe, Lrp1*) (28). *In vivo* two-photon microscopy on open cranial window of immunocompetent Cx3cr1^GFP/WT^; Ccr2^RFP/WT^ reporter mice revealed that resident microglia (that strongly expressed GFP) accumulate in clusters at the tumor periphery and are stationary while newly infiltrated MDM are highly mobile (29). Using the GL261 model and scRNA-seq of myeloid cells (CD11b^+^), Ochocka and colleagues found that in healthy brains, microglia represented the vast majority of myeloid cells (90%) while BAMs constituted only ~6% (19). In tumor-bearing mice, microglia (Tmem119^+^) remained dominant, constituting ~ 65% of all myeloid cells, but were displaced from the tumor core by infiltrating MDMs (Gal-3^+^) (19). These results were corroborated by Banerjee and colleagues who reported the spatiotemporal distribution of microglia and MDMs at early (14–15 days post-injection [dpi]), intermediate (24–25 dpi), and terminal (28–36 dpi) phases of glioma using Cx3cr1^CreER/+^: R26^tdT/+^ mice and two syngeneic mouse glioma models (GL261 and CT-2A). Their results showed that microglia accumulated inside the tumor at the early phase of tumorigenesis but were driven out of the tumor at later times by MDMs, demonstrating competition between these two macrophage sub-populations (30) (**Figure 2**). This was further observed by De Leo et al. using another murine glioma model, namely SB28, where MDMs constituted >75% of all F4/80^+^ brain/glioma macrophages and in which neutralization of MDMs with anti-CD49d antibodies led to a surge in intra-tumoral microglia frequencies (31). MDM are not prevalent in tumors of patients with newly diagnosed GB, accounting for <20% of all macrophages, but constitute ~50% of all GAMs in recurrent GB (31), pointing to distinct TMEs in recurrence. Antunes and colleagues extended GAM profiling to identify differences between primary and recurrent tumors from human and mouse cohorts (18). Their data identified *SALL1, TMEM119, P2RY12* as markers of microglia-derived GAMs that dominated the TME of primary tumors and *TGFBI, CLEC12A* and *FXYD5* as markers of MDMs that outnumbered microglia in recurrence and were enriched at pimonidazole (PIMO)-positive hypoxic tumor regions (**Figure 2**). Both microglia and MDMs presented significantly higher pro-angiogenic activity compared to normal brain microglia from control mice, as revealed using culture on chicken chorioallantoic membrane. In addition, they were incapable of inducing allogeneic proliferation of CD4^+^ or CD8^+^ T lymphocytes *ex vivo*, potentially due to an immunosuppressed state. Yeo et al. further detailed the immune changes occurring with GB progression particularly in an *EGFR*-driven GB mouse model (32). Their results showed that early GB tumors were mainly composed of pro-inflammatory microglia with expression of complement (*C1qb, C1qa, C1qc*) and lipoprotein catabolic enzymes (*Apoe, Ctsd*) genes, in contrast to late GB tumors that were populated by immunosuppressive pro-tumoral macrophages. It is important to note that not all GAMs are deleterious. Using scRNA-seq in two orthotopic glioma models (GL261 and CT-2A) and in a genetically engineered mouse glioma model (*EGFRvIII+/TP53-/PTEN-)*, Kim and colleagues identified CD169^+^ (*SIGLEC1*) MDMs, induced by IFN-γ, as a beneficial GAM subset contributing to antitumor immunity (33). They showed that blockade of CD169 on bone marrow-derived macrophages (BMDMs) *in vitro* blunted their phagocytic and immunostimulatory capacities (33).

The TME of GB is lymphocyte poor, consistent with the reported resistance of GB patients to immune checkpoint blockade (34–36). In contrast, brain metastases (BrM) originating from various non-CNS cancers, such as melanoma, lung or breast cancer exhibit higher proportions of recruited T and B lymphocytes (37, 38). A recent analysis of >800 GB patients tumors identified 3 TME subtypes (TME low, med, or high) (39). Notably, TME high MES class tumors are enriched in T cell activation and exhaustion markers, regulatory T cells, and expression of immune checkpoints and associated with a trend towards improved survival in clinical trials testing neoadjuvant/adjuvant anti-PD-1 treatment or oncolytic virus PSVRIPO therapy (39). Interrogation of the Glioma Longitudinal AnalySiS (GLASS) cohort further revealed TME transitions with GB progression (39). Collectively, the fine mapping of the GB TME has revealed the heterogeneity of GAM phenotypes according to the glioma type, stage and treatment and have unraveled subsets of potential clinical impact. We posit that a 3D holistic view of the entire tumor (40) will localize the identified GAM subsets in space, define the molecular basis of their heterogeneity and ultimately lead to new approaches to overcome therapeutic failures in GB (41–44).

### 3.2 Metabolic regulation of myeloid cells in the GB TME

Spatial profiling of human GB showed distinct immune landscapes in the tumor peri-vascular (PVZ) versus peri-necrotic (PNZ) zones, which correlated with patient survival (15), highlighting the impact of metabolic gradients in shaping anti-tumor immunity. Disrupting the metabolic symbiosis between the tumor and its TME is thus a promising area for therapeutic intervention. In GB, metabolic adaptations can partly explain the impressive plasticity of GAMs. Current research in this area is focused on characterizing metabolic effectors that impact GAM diversity and functions in immunosuppression, tumor progression and clinical prognosis (45, 46). A notable example is the enzyme arginase 1 (Arg 1) expressed in GAMs that hydrolyzes L-arginine into urea and L-ornithine. Arg 1 mediates immune evasion by depleting L-arginine and depriving effector T cells and natural killer (NK) cells that need this nutrient for their proliferation (47). In GB, Arg 1 inhibition using pegylated arginine deiminase (ADI-PEG20) improved radiotherapy efficacy in immunocompetent orthotopic mouse models and promoted GAM differentiation into a pro-inflammatory anti-tumoral phenotype (48). The immunomodulatory role of Arg 1 is thought to be mediated by nitric oxide (NO), which limits mitochondrial metabolism by inhibiting the activity of acotinase 2 (A2) and pyruvate dehydrogenase (PDH), driving macrophages into a glycolytic state (49). De Leo et al. showed that ER stress-induced activation of protein kinase R (PKR)-like endoplasmic reticulum kinase (PERK) promoted enhanced glycolysis in MDMs through upregulation of the glucose transporter GLUT1. They showed that MDM-immunosuppressive function was partly mediated by IL-10, which was induced by lactylation downstream of lactate intracellular accumulation (31). To survive in the GB hypoxic and acidic TME, GAMs also engage the arginine-ornithine-polyamine axis to produce the highly basic polyamine that buffers their intracellular pH. Blockade of polyamine biosynthesis proved efficient in GAM depletion and reprogramming leading to improved OS in glioma-bearing mice (50). In parallel, GAMs upregulate the expression of Na/H1 exchange protein (*NHE1*) to export excess protons to the extracellular space. Hasan et al. demonstrated that selective deletion of *NHE1* in Cx3cr1^+^ myeloid cells led to an immunogenic TME particularly in response to TMZ (51). GAMs surrounding the peri-necrotic hypoxic regions of the tumor express the creatine enzyme arginine-glycine amidinotransferase and produce creatine from arginine. Using isotopic tracing, Rashidi et al. showed that secreted creatine is taken up by tumor cells expressing the creatine transporter SLC6A8 and that pharmacological inhibition of this transporter slowed tumor growth (52). Tryptophan metabolism to kynurenine provides an additional axis governing the immunosuppressive GB TME. Kynurenine produced by tumor cells activates the aryl hydrocarbon receptor (AHR) in GAMs. AHR induces *CCR2* expression, which furthers GAM recruitment, and upregulates *CD39* that promotes adenosine-dependent T cell dysfunction (53). Tryptophan metabolism also results in the accumulation of low concentrations (<100 nM) of quinolate (QA), nduces the activation of the N-methyl-D-aspartate (NMDA) receptor and the Foxo1/PPARγ pathway that induces the differentiation of highly immunosuppressive macrophages (54). Pires‐Afonso et al. uncovered aconitate decarboxylase ACOD1 (*Irg1*) as an additional metabolic effector involved in this process. Compared to control mice, *Acod1*-deficient mice exhibited GAMs with increased immune reactivity and had reduced tumor load (55). Collectively, these studies allow better understanding of how GAM spatial distribution according to metabolite gradients affects their phenotypic and functional heterogeneity. Metabolic effectors thus provide new therapeutic strategies and perspectives in the treatment of GB.

### 3.3 Myeloid cells-glioma cells crosstalk in GB

Several examples illustrate the tight dialogue between GAMs and GB phenotypes. On one hand, glioma programs e.g. regulated epigenetically by SETD2, SOX10, CLOCK and KDM6 instruct GAM landscapes. Liu et al. showed that deletion or nonsense mutations in the histone methyltransferase gene *SETD2* were associated with increased infiltration of GAMs mediated by TGFβ signaling (56). Wu et al. demonstrated that loss of the chromatin regulator *SOX10*, particularly in the receptor tyrosine kinase I (RTK-I) glioma tumor subtype, resulted in NPC- to MES-like transition, associated with GAM recruitment (57), and Xuan et al. linked glioma *CLOCK* expression to microglia intra-tumoral infiltration through the Olfactomedin-like 3 (OLFML3)/HIF1α/Legumain (LGMN)/P-selectin glycoprotein ligand-1 (PSGL-1) axis (58).

On the other hand, myeloid-derived signals shape glioma states. For instance, Rao et al. described a differential requirement of CSF-1R signaling in *PDGFB*-induced NPC-like versus in *HRAS*-driven gliomas (59) and the IL-1β pathway was shown to promote *PDGFB*-driven GB, through induction of monocyte chemoattractant protein (MCP)1-mediated recruitment of MDMs (60). Zhang et al. demonstrated that TREM1 expression on CD163^+^ GAMs is central in GB TME remodeling (61). This was corroborated by Dong et al. who reported that hypoxia-inducible factor (HIF)1α-dependent induction of TREM1 on GAMs promoted MES-like state transition via TGFβ secretion (62). Goswami et al. showed that lysine-specific demethylase 6B (*KDM6B*) promoted the expression of key immunosuppressive factors in GAMs, including signal-regulatory protein α *(Sirpa)*, suppressor of cytokine signaling 3 *(Socs3)*, and v-Maf avian musculoaponeurotic fibrosarcoma oncogene homolog B *(Mafb)* (63), and that myeloid-specific ablation of *KDM6B* reprogrammed GAMs into anti-tumoral effectors, improving the efficacy of anti-PD1 treatment in glioma-bearing mice (34). Altogether, these studies illustrate the complex mechanisms underlying GSC-myeloid cell reciprocal adaptations to intrinsic and extrinsic stresses in the tumor, which drive clonal selection, ITH and therapeutic resistance.

## 4 Myeloid-based clinical trials in patients with glioblastoma

**Figure 3:**
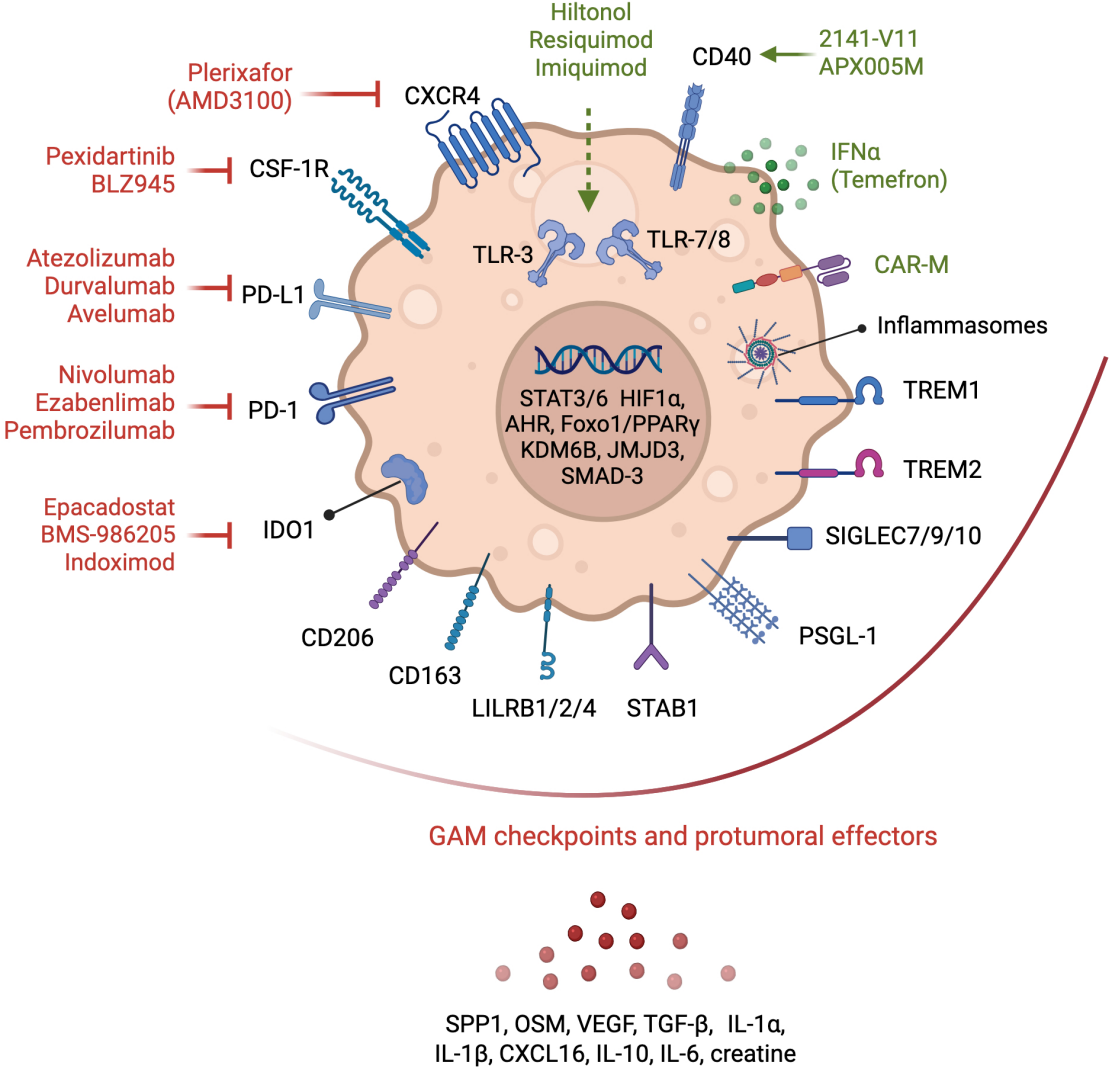
Glioma-associated macrophages therapeutic targets. Myeloid-based strategies evaluated in clinical trials in patients with GB are depicted. Antagonists of chemokine receptors involved in myeloid cell recruitment, and of immune checkpoints and metabolic effectors that contribute to the pro-tumoral functions of GAMs are shown in red. Immunostimulatory approaches, including CD40 and TLR agonists, and myeloid-based cell therapies, such as chimeric antigen receptor (CAR)-expressing macrophages (CAR-M) or myeloid cells engineered to express IFNα2 (Temefron), are illustrated in green. Myeloid checkpoints, demonstrated preclinically to contribute to tumorigenesis and immunosuppression, but await clinical testing in GB, are shown under the red arc. GAM transcriptional regulators and secreted factors important in tumor growth and TME remodeling are also illustrated.

A census of clinicaltrials.gov in July 2024 with the keywords “glioblastoma” and “immune” retrieves 324 studies testing different immunomodulatory strategies in patients with glioblastoma. Of these, we summarize 54 trials involving myeloid-based approaches (**Table 1**). These can be grouped under six main strategies namely, 1) immune checkpoint inhibitors (ICI), primarily anti-PD-1 and anti-PD-L1; 2) CSF-1R inhibitors, 3) myeloid depletion/exclusion, 4) immunostimulatory approaches, namely (i) CD40 agonistic antibodies, (ii) controlled human IL-12 gene therapy and (iii) Toll-like receptor (TLR) agonists, 5) macrophage-based cell therapy, and 6) metabolic checkpoint inhibitors (**Table 1**, **Figure 3**). Of the 54 listed studies, 18 have reported results but only three have completed phase III clinical trials in patients with GB, namely CheckMate 143 (34), CheckMate 498 (36), and CheckMate 548 (35), which evaluated the anti-PD-1 antibody nivolumab as a single agent, or in combination with RT or with RT plus TMZ, respectively, as detailed below.

**Table 1:** Selected clinical trials evaluating macrophage-based strategies in GB.

Strategy	Sponsor	Other drug	Condition	Phase	Status	NCT number
Immune checkpoint inhibitors						
Nivolumab (anti-PD-1)	Bristol-Myers Squibb	N/A	Recurrent GB	Phase III	**Completed (with results)**	NCT02017717
Nivolumab (anti-PD-1)	Bristol-Myers Squibb	RT	Newly diagnosed unmethylated MGMT GB (CheckMate 498)	Phase III	**Completed (with results)**	NCT02617589
Nivolumab (anti-PD-1)	Bristol-Myers Squibb	RT/TMZ	Newly diagnosed MGMT-methylated GB	Phase III	**Completed (with results)**	NCT02667587
Nivolumab (anti-PD-1)	National Cancer Institute (NCI)	Relatlimab (anti-LAG-3)	Recurrent GB	Phase II	Not yet recruiting	NCT06325683
Nivolumab (anti-PD-1)	Sidney Kimmel Comprehensive Cancer Center at Johns Hopkins	Urelumab (anti-LAG-3)	Recurrent GB	Phase I	Completed	NCT02658981
Nivolumab (anti-PD-1)	National Cancer Institute (NCI)	Ipilimumab (anti-CTLA4)/TMZ	Newly Diagnosed GB	Phase I	Completed	NCT02311920
Nivolumab (anti-PD-1)	Bart Neyns, Universitair Ziekenhuis Brussel	Intra-tumoral Ipilimumab (anti-CTLA4)	Following the Resection of Recurrent GB	Phase I	Unknown status	NCT03233152
Ezabenlimab (anti-PD-1)	Johann Wolfgang Goethe University Hospital	NK-92/5.28.z (anti-HER2-CAR-NK)	Recurrent HER2-positive GB	Phase I	Active, not recruiting	NCT03383978
Pembrolizumab (anti-PD-1)	University of Florida	TTF, TMZ	GB	Phase II	**Active, not recruiting (with results)**	NCT03405792
Pembrolizumab (anti-PD-1)	Istari Oncology, Inc.	Lerapolturev (PVSRIPO)	GB	Phase II	Active, not recruiting	NCT04479241
Pembrolizumab (anti-PD-1)	University of Louisville	TMZ	GB	Phase I	Recruiting	NCT05700955
Pembrolizumab (anti-PD-1)	National Cancer Institute (NCI)	N/A	Younger patients with r/r high-grade gliomas	Phase I	Recruiting	NCT02359565
Durvalumab (anti-PD-L1)	Northwestern University	Tremelimumab (anti-CTLA4)	Recurrent GB	Phase II	**Completed (with results)**	NCT02794883
Avelumab (anti-PD-L1)	Clinique Neuro-Outaouais	RT/TMZ	GB	Phase II	Completed	NCT03047473
Avelumab (anti-PD-L1)	Vaximm GmbH	VXM01 (T cell vaccine against VEGFR2)	Recurrent GB	Phase I-II	Active, not recruiting	NCT03750071
Atezolizumab (anti-PD-L1)	M.D. Anderson Cancer Center	Cabozantinib (TKI)	Recurrent GB	Phase I-II	Recruiting	NCT05039281
Atezolizumab (anti-PD-L1)	Stony Brook University	RT	GB	Early Phase 1	Recruiting	NCT05423210
Atezolizumab (anti-PD-L1)	M.D. Anderson Cancer Center	RT/TMZ	GB	Phase I-II	Active, not recruiting	NCT03174197
Atezolizumab (anti-PD-L1)	National Cancer Institute (NCI)	Tiragolumab (anti-TIGIT)	Recurrent GB	Phase II	Not yet recruiting	NCT06328036
CSF-1R inhibitors						
Pexidartinib (PLX-3397)	Daiichi Sankyo	N/A	Recurrent GB	Phase II	**Terminated (with results)**	NCT01349036
Pexidartinib (PLX-3397)	Daiichi Sankyo	RT/TMZ	Newly Diagnosed GB	Phase I-II	**Completed (with results)**	NCT01790503
BLZ945	Novartis	spartalizumab (anti-PD-1)	Advanced Solid Tumors (r/r GB)	Phase I-II	**Terminated (with results)**	NCT02829723
Depletion/exclusion of myeloid cells						
Capecitabine	Case Comprehensive Cancer Center	Bevacizumab (anti-VEGF)	Recurrent GB	Phase I	Active, not recruiting	NCT02669173
Plerixafor (AMD3100) (CXCR4 antagonist)	Lawrence D Recht	RT/TMZ	Newly diagnosed high grade gliomas	Phase II	**Completed (with results)**	NCT01977677
Plerixafor (AMD3100) (CXCR4 antagonist)	Lawrence D Recht	RT/TMZ	GB	Phase II	**Active, not recruiting (with results)**	NCT03746080
Plerixafor (AMD3100) (CXCR4 antagonist)	Fred Hutchinson Cancer Center	Autolgous HSCT/TMZ + O6-benzylguanine (MGMT inhibitor)	Malignant Gliomas	Phase I-II	**Terminated (with results)**	NCT00669669
Plerixafor (AMD3100) (CXCR4 antagonist)	Patrick Y. Wen	Bevacizumab (anti-VEGF)	Recurrent high-grade gliomas	Phase I	Terminated	NCT01339039
Immunostimulatory - CD40 agonists, IL-12 gene therapy						
2141-V11 (anti-CD40 agonist)	Darell Bigner	D2C7-IT (EGFR- immunotoxin)	Recurrent malignant glioma	Phase I	Recruiting	NCT04547777
APX005M (sotigalimab) (anti-CD40 agonist)	Pediatric Brain Tumor Consortium	N/A	CNS tumors (GB)	Phase I	Active, not recruiting	NCT03389802
Ad-RTS-hIL-12 (Veledimex-regulatable IL-12 gene therapy)	Alaunos Therapeutics	Veledimex (activator of RTS), Cemiplimab-Rwlc (anti-PD-1)	GB	Phase II	Completed	NCT04006119
Ad-RTS-hIL-12 (Veledimex-regulatable IL-12 gene therapy)	Alaunos Therapeutics	Veledimex (activator of RTS), Nivolumab (anti-PD-1)	GB	Phase I	Completed	NCT03636477
Ad-RTS-hIL-12 (Veledimex-regulatable IL-12 gene therapy)	Alaunos Therapeutics	N/A	Recurrent GB	Phase I	Completed	NCT02026271
Immunostimulatory - TLR agonists (Vaccines)						
Hiltonol (Poly-ICLC - TLR3 agonist)	Sidney Kimmel Comprehensive Cancer Center at Johns Hopkins	N/A	Brain and CNS tumors	Phase II	**Terminated (with results)**	NCT00052715
Hiltonol (Poly-ICLC - TLR3 agonist)	Sidney Kimmel Comprehensive Cancer Center at Johns Hopkins	RT/TMZ	Newly diagnosed GB	Phase II	**Completed (with results)**	NCT00262730
Hiltonol (Poly-ICLC - TLR3 agonist)	NYU Langone Health	RT/TMZ	Recurrent GB	Phase II	**Completed (with results)**	NCT00262730
Hiltonol (Poly-ICLC - TLR3 agonist)/Imiquimod (TLR7 agonist)	Stemline Therapeutics, Inc.	SL-701 (peptide vaccine against IL-13Ra2, EphA2, and survivin)/Bevacizumab (anti-VEGF)/GM-CSF	Recurrent GB	Phase I-II	**Completed (with results)**	NCT02078648
Hiltonol (Poly-ICLC - TLR3 agonist)	University Hospital, Geneva	IMA950 (Tumor-associated peptide vaccine)/TMZ	Newly diagnosed HLA-A2 GB	Phase I-II	Completed	NCT01920191
Hiltonol (Poly-ICLC - TLR3 agonist)/Resiquimod (TLR7/8 agonist)	Jonsson Comprehensive Cancer Center	Autologous tumor lysate-pulsed DC vaccine	Brain tumors	Phase II	Active, not recruiting	NCT01204684
Hiltonol (Poly-ICLC - TLR3 agonist)	University Hospital, Geneva	IMA950 (Tumor-associated peptide vaccine)/Pembrolizumab (anti-PD-1)	Relapsing glioblastoma irrespective of MGMT and IDH gene status	Phase I-II	Active, not recruiting	NCT03665545
Hiltonol (Poly-ICLC - TLR3 agonist)	Immatics Biotechnologies GmbH	APVAC1 or APVAC2 (unmutated and neoantigens peptive vaccine)/RT/TMZ, GM-CSF	Newly diagnosed GB	Phase I	Completed	NCT02149225
Hiltonol (Poly-ICLC - TLR3 agonist)	Mustafa Khasraw, MBChB, MD, FRCP, FRACP	P30-EPS (P30-linked EphA2/CMV pp65/survivin peptide vaccine)	Newly diagnosed, unmethylated, and untreated GB	Phase Ib	Recruiting	NCT05283109
Hiltonol (Poly-ICLC - TLR3 agonist)	Albert Einstein College of Medicine	Personalized peptide vaccine/TTF	Glioblastoma	Phase Ia-Ib	Active, not recruiting	NCT03223103
Hiltonol (Poly-ICLC - TLR3 agonist)	Jonsson Comprehensive Cancer Center	ALT-DC (autologous tumor lysate-pulsed DC vaccine)/Pembrolizumab (anti-PD-1)	Recurrent Glioblastoma	Phase I	Recruiting	NCT04201873
Hiltonol (Poly-ICLC - TLR3 agonist)	Washington University School of Medicine	Neoepitope-based personalized vaccine/TMZ	Newly diagnosed GB	Phase I	Terminated	NCT02510950
Hiltonol (Poly-ICLC - TLR3 agonist)	Washington University School of Medicine	NeoVax/Nivolumab (anti-PD-1)/Ipilumumab (anti-CTLA4)	Newly diagnosed unmethylated GB	Phase I	Terminated	NCT03422094
Hiltonol (Poly-ICLC - TLR3 agonist)	Shanghai 10th People’s Hospital	NeoPep vaccine 1 and 2	Newly diagnosed GB	Unknown	Recruiting	NCT05557240
Macrophage-based cell therapy						
Temefron (Autologous CD34^+^-enriched HSPCs genetically modified with human Interferon-α2)	Genenta Science	N/A	Unmethylated MGMT GB	Phase I-IIa	Recruiting	NCT03866109
Anti-HER2 CAR macrophages (CT-0508)	Carisma Therapeutics Inc	N/A	HER2 overexpressing solid tumors	Phase I	Active, not recruiting	NCT04660929
Metabolic checkpoint inhibitors						
Epacadostat (IDO1 Inhibitor)	Incyte Corporation	nivolumab (anti-PD-1)	Select advanced cancers including brain tumors	Phase I-II	**Completed (with results)**	NCT02327078
Epacadostat (IDO1 Inhibitor)	Washington University School of Medicine	RT/retifanlimab (anti-PD-1)/Bevacizumab (anti-VEGF)	Recurrent GB	Phase II	Active, not recruiting	NCT03532295
BMS-986205 (IDO1 Inhibitor)	Northwestern University	Nivolumab (anti-PD-1)	GB	Phase I	Active, not recruiting	NCT04047706
Indoximod (IDO1 Inhibitor)	NewLink Genetics Corporation	RT/TMZ	GB	Phase I-II	**Completed (with results)**	NCT02052648
PF-06840003 (IDO1 Inhibitor)	Pfizer	N/A	GB	Phase I	**Terminated (with results)**	NCT02764151

In bold are clinical trials whose status is terminated/completed (with results).

### 4.1 Immune checkpoint inhibitors

Besides their effects on lymphocytes, both PD-1 and PD-L1 have been demonstrated to modulate macrophage survival, proliferation and activation. PD-L1 regulates macrophage activation and proliferation (64) and PD-1 expression by macrophages inhibits their anti-tumoral phagocytic activity (65–67). High-dimensional analysis of the intra-tumoral immune remodeling elicited by anti-PD-1/CTLA-4, revealed important contributions of the myeloid compartment in therapeutic resistance (68) through GAM reprogramming (69, 70). Despite ICI approval in over 14 cancer indications, and their efficacy in durable responses in several advanced solid tumors (71), the three phase III clinical trials in GB had disappointing results. CheckMate 143 compared nivolumab to bevacizumab in recurrent GB at first recurrence and found comparable median OS between the two treatments (34). CheckMate 498 tested nivolumab in combination with RT in newly diagnosed GB with unmethylated MGMT promoter. The study did not meet the primary endpoint of improved OS and found that nivolumab + RT demonstrated shorter OS than TMZ + RT (36). CheckMate 548 reported similarly disappointing results of the evaluation of nivolumab in combination with TMZ and RT in newly diagnosed GB with methylated MGMT promoter (35). The U.S. Food and Drug Administration (FDA) has granted approval of the anti-PD-1 pembrolizumab (Keytruda^®^) for certain adult and pediatric patients with advanced solid tumors that have high microsatellite instability (MSI-H), DNA mismatch repair deficiency (dMMR), or high tumor mutational burden (TMB-H) in a tumor agnostic manner. This approval was based on results of phase 2 trials i.e., KEYNOTE-158 (NCT02628067), KEYNOTE-164 (NCT02460198), and KEYNOTE-051 (NCT02332668) that showed the efficacy of pembrolizumab in this indication in 504 patients across over 30 cancer types. A phase I trial (NCT02359565) is currently evaluating the safety and preliminary efficacy of pembrolizumab in children and young adult patients with recurrent, progressive, or refractory high-grade gliomas, diffuse intrinsic pontine gliomas, hypermutated brain tumors, ependymoma or medulloblastoma. Ongoing trials evaluating ICIs in GB are testing safety and efficacy of nivolumab in combination with the anti-CTLA-4 antibody ipilimumab, administered either intravenously (i.v.) (NCT02311920) or intra-tumorally (i.t.) (NCT02017717), or in combination with the anti-LAG3 antibodies urelumab (NCT02658981) or relatlimab (NCT06325683). Other clinical trials are testing anti-PD-L1 antibodies, i.e., *a)* durvalumab in combination with the anti-CTLA-4 antibody tremelimumab in recurrent GB (NCT02794883), *b)* atezolizumab in combination with RT (NCT05423210), RT plus TMZ (NCT03174197) or with the anti-TIGIT antibody tiragolumab (NCT06328036) and *c)* avelumab in combination with standard-of-care (NCT03047473). The latter reported their findings in (72). In this phase II monocentric study, 30 patients with newly diagnosed GB were treated with avelumab at 10 mg/kg (i.v.) concurrent with TMZ. No new safety signals were induced by the combination, however addition of avelumab to standard-of-care did not show apparent benefit in improving OS. Besides ICI combinations, immune checkpoint blockade may also increase the efficacy of other targeted therapies including other immunotherapy modalities. Several ongoing studies are testing such ICI combinations e.g. of atezolizumab with the tyrosine kinase inhibitor (TKI) cabozantinib in recurrent GB (NCT05039281), the anti-PD-1 ezabenlimab with a HER2-CAR-NK (NK-92/5.28.z) in recurrent HER2-positive GB (NCT03383978), avelumab with a T cell vaccine against VEGFR2 (VXM01) (NCT03750071) or pembrolizumab with tumor treating fields (TTF) therapy (NCT03405792) or with the oncolytic virus lerapolturev (formerly PVSRIPO) (NCT04479241).

### 4.2 CSF-1R inhibitors

CSF-1R blockade is an attractive therapeutic option aimed at inhibiting the recruitment and pro-tumoral reprogramming of GAMs (73). In preclinical models of GB, CSF-1R inhibition had opposite effects on tumor growth depending on the tumor driver oncogene. In a PDGFB-driven glioma model, CSF-1R blockade inhibited tumor growth and improved mouse survival by re-programming GAMs rather than causing their depletion (59, 74). In contrast, in a RAS-driven model, CSF-1R depletion accelerated tumor growth (75). Two CSF-1R inhibitors have been evaluated in patients with GB in early phase trials, namely BLZ945 (in combination with anti-PD-1 - NCT02829723) and Pexidartinib (PLX-3397), either as a monotherapy (NCT01349036) (76) or in combination with RT plus TMZ (NCT01790503) (**Figure 3**). The results of the phase II study testing PLX3397 as a monotherapy in recurrent GB showed that it crossed the BBB and attenuated CD14^dim^/CD16^+^ monocytes in plasma but did not show efficacy in the 37-patient study cohort (76). Similarly, no improvement was attained by combining this inhibitor with RT plus TMZ (NCT01790503). In a phase I-II study assessing BLZ945 safety and preliminary efficacy as a monotherapy or in combination with the anti-PD-1 antibody spartalizumab (PDR001) in 146 patients with advanced/metastatic solid tumors including in 29 patients with GB, limited efficacy was reported particularly in non-MES GB patients (77), as determined by a correlation between PDGFRA gene expression and best percent change in tumor size. This was associated with decreased non-classical and intermediate monocytes in peripheral blood mononuclear cells (PBMCs) after treatment at multiple doses of BLZ945 and a downregulation of a macrophage geneset in the TME in tumor biopsy samples. Together, these results suggest that different GB subtypes might present differential responsiveness to CSF-1R inhibition.

### 4.3 Myeloid cell depletion/exclusion

Preclinical studies have shown that low dose chemotherapy depletes myeloid-derived suppressor cells (MDSC) (78–80). An early phase 0/I trial evaluated the effect of neoadjuvant treatment with low-dose capecitabine as a strategy to deplete MDSC in patients with recurrent GB, followed by capecitabine in combination with bevacizumab (NCT02669173). In 11 patients evaluated, the treatment was tolerated and led to a reduction in circulating MDSC countered by enhanced intra-tumoral infiltration of cytotoxic lymphocytes (81). An alternative approach is to restrict monocyte recruitment and to abrogate MDM-mediated tumor revascularization post RT (82, 83). Two clinical trials in patients with GB evaluated blocking CXCR4-stromal cell-derived factor-1 (SDF-1/CXCL12 interaction using the CXCR4 antagonist plerixafor (AMD3100) (NCT03746080, NCT01977677) (84). Plerixafor was also tested in combination with bevacizumab (NCT01339039) or with hematopoietic stem cell transplantation to improve chemotherapy tolerance (NCT00669669) (85). Promising results from these trials have been reported (84, 85) that warrant further testing of CXCR4 blockade in larger trials.

### 4.4 Immunostimulatory approaches

#### 4.4.1 Anti-CD40 agonistic antibodies

CD40, a member of the tumor necrosis factor (TNF) receptor family, is expressed on antigen-presenting cells (APC), including microglia and macrophages (86) and is involved in their activation towards an anti-tumoral profile. Agonistic anti-CD40 antibodies mimic the actions of CD40 ligand (CD40L) in enhancing antigen processing and presentation by APC leading to T cell stimulation, and in inducing the production of downstream macrophage effectors, e.g., reactive oxygen and nitrogen species, pro-inflammatory cytokines, and the CD4^+^ T cell chemokine CCL5 required for immune checkpoint blockade efficacy (87). Several preclinical studies have demonstrated the value of anti-CD40 agonist treatment in reprogramming GAMs and boosting anti-tumor immunity, e.g. in combination with CSF-1R blockade (88), IL-6 inhibition (89) or with the microtubule-disrupting agent lisavanbulin (BAL101553) in treating ICI-resistant glioma (90). Currently, there are two ongoing phase I clinical trials evaluating anti-CD40 agonist antibodies in GB, namely, APX005M (sotigalimab) (NCT03389802) and 2141-V11 in combination with D2C7-IT, a dual-specific immunotoxin targeting wild-type EGFR and mutant EGFR variant III (EGFRvIII) (NCT04547777) (**Figure 3**). The latter is based on encouraging pre-clinical results in orthotopic glioma mouse models revealing that D2C7-IT plus anti-CD40 agonist treatment elicited an anti-tumoral phenotype of macrophages and microglia and drove an effective CD8^+^ T cell response (91).

#### 4.4.2 Controlled human IL-12 gene therapy

IL-12, an innate cytokine produced by activated APC, is a central inducer of interferon (IFN) γ and governs the activation of both innate and adaptive lymphocytes. To counter reported toxicity induced by systemic IL-12, Veledimex (VDX)-controlled intra-tumoral induction of IL-12 from an adenoviral vector, using the RheoSwitch Therapeutic System^®^ (RTS^®^), has been tested in GB in phase I and II studies, both as a monotherapy and in combination with anti-PD-1 (NCT02026271; NCT03636477; NCT04006119). The results from the phase I studies showed that IL-12 induced CD8^+^ T cells intratumoral infiltration, induction of immune checkpoint signaling, and amelioration in survival (92). The results of the phase II trial are pending.

#### 4.4.3 TLR ligands

TLR ligands have long been used to stimulate macrophages and overcome their immunosuppressive function in tumors. For instance, Bacillus Calmette- Guérin (BCG) that stimulates TLR2 is used in the clinic to treat patients with bladder cancer (93). Engagement of endosomal TLRs, i.e. TLR3, 7, 8 and 9, induces not only a pro-inflammatory response but also a type I IFN anti-tumoral immune response. At least 14 clinical trials are testing the TLR3 agonist poly-ICLC complexed with carboxy-methyl-cellulose (Hiltonol) in patients with GB, mainly in combination with synthetic peptide-, tumor lysate-, or cell-based anti-cancer vaccines (94, 95) (**Table 1**). The TLR7 agonist imiquimod, approved by the FDA for squamous and basal cell carcinoma via topical application, and the TLR7/8 agonist resiquimod are also being tested in GB, but less so than Hiltonol (**Table 1**, **Figure 3**). Preclinical studies are refining the modality of TLR agonist administration, encapsulating them in nanoparticle formulations for intratumoral and intravenous administration. For instance, Turco et al. have recently reported that encapsulated R848, a TLR7/8 agonist, administered i.v., led to experimental glioma eradication independently of T cells via macrophage reprogramming (96). Another approach to deliver agonists for TLRs (or other pattern recognition receptors (PRR)) is through bacteriotherapy. Zhang et al. demonstrated that an attenuated *Salmonella-*based bacterium-hydrogel nano-capsules elicited glioma cell pyroptosis and anti-tumor immunity (97). A TLR9 ligand formulated in a virus-like particle have demonstrated promising results in patients with metastatic melanoma in overcoming resistance to anti-PD-1 (98), but has not yet been evaluated in CNS tumors.

### 4.5 Macrophage-based cell therapy

Taken the massive GAM infiltration in the GB TME, arming monocytes and macrophages with therapeutic agents, e.g., cytokines such as IFNα, or an engineered chimeric antigen receptor (CAR), is a promising therapeutic approach. An ongoing phase I-IIa clinical trial (NCT03866109) is currently recruiting patients with unmethylated *MGMTp* GB to evaluate the safety and preliminary efficacy of Temefron, an autologous CD34^+^-enriched HSPCs genetically modified to express human IFNα2 under the Tie2 promoter. This is based on the high tumor-homing property of Tie2^+^ monocytes and promising preclinical data by De Palma et al., showing improved anti-tumor immunity and reduced tumor angiogenesis using this approach (99). Contrary to limited intra-tumoral accumulation of CAR-T cells, the significant infiltration of GAMs in the TME also supports the rationale for CAR-macrophages (CAR-M) development. An active trial in HER2-overexpressing solid tumors will be testing anti-HER2 CAR-M (CT-0508) alone or in combination with pembrolizumab (NCT04660929) (**Figure 3**).

### 4.6 Metabolic checkpoint inhibitors

As discussed above, GAMs rewire their metabolism to survive the harsh metabolic tumor environment that is hypoxic, acidic and nutrient-depleted. Through metabolic adaptations, GAMs further TME immunosuppression e.g., by depriving effector T cells of tryptophan via the activity of Indoleamine 2,3-Dioxygenase 1 (IDO1). IDO1 blockade has been evaluated in several clinical trials in patients with solid tumors with contrasting results. Four IDO1 inhibitors have been evaluated in GB in early phase studies, namely epacadostat, BMS-986205, indoximod and PF-06840003 (**Table 1**, **Figure 3**). Epacadostat (100) and BMS-986205 were tested in combination with nivolumab (NCT02327078; NCT04047706). Epacadostat was also evaluated in combination with retifanlimab (anti-PD-1), RT plus bevacizumab (NCT03532295). Indoximod was tested in combination with TMZ (NCT02052648) and PF-06840003 as a single agent (NCT02764151). However, the disappointing results (101) from a randomized phase III clinical trial (ECHO301) evaluating epacadostat in combination with pembrolizumab in patients with metastatic melanoma of (NCT02752074) has dampened enthusiasm for IDO1 inhibitor development (102). Such failure could stem from compensatory mechanisms by the additional tryptophan metabolism enzymes IDO2 and tryptophan 2,3- dioxygenase (TDO).

### 4.7 Suspended macrophage-based approaches

Blockade of the phagocytosis inhibitor CD47 with the antibody magrolimab has demonstrated futility with an increased risk of death in patients with acute myeloid leukemia (AML) (phase III trial ENHANCE III - NCT05079230). The FDA has requested a full clinical hold on all magrolimab studies in AML and myelodysplastic syndromes (MDS), and Gilead has since February 2024 paused all ELEVATE studies testing magrolimab in solid tumors. A phase I study testing this agent in children and adults with recurrent or progressive malignant brain tumors (NCT05169944) has also been suspended. CD47 is overexpressed on cancer cells and provides a “don’t eat me” signal by binding to signal regulatory protein α (SIRPα) on macrophages leading to inhibition of phagocytosis. Antibodies targeting CD47 release this inhibitory signal and bind to the Fc receptor on macrophages providing a concurrent “eat me signal” needed for the macrophage killing activity. Since CD47 is ubiquitously expressed on normal human cells, the reported safety issues with magrolimab might be related to on-target toxicity. Alternative approaches are currently testing anti-CD47 agents with mutated Fc (ALX148) or SIRPα decoys (e.g., TTI-622) fused to IgG4 Fc with a weaker killing activity. Bi-specific antibodies that dually recognize a tumor-associated antigen (e.g., CD19, CD20, PD-L1, EGFR) and CD47 can enhance specificity and limit toxicity, and are being evaluated in hematologic and solid tumors, in preclinical (103–105) and clinical (NCT03804996; NCT04806035) studies but testing in brain malignancies is not yet reported.

## 5 Outlooks: rational macrophage-based therapeutic strategies

To date targeting macrophages with non-discriminatory methods e.g. with CSF-1R inhibition has failed to demonstrate clinical benefit. Similarly, inhibition of the CC-chemokine ligand 2 (CCL2) did not demonstrate anti-tumoral effects, e.g., in a phase II clinical trial of Carlumab (CNTO 888) in patients with metastatic castration-resistant prostate cancer (106). Dual blockade of CCR2/CCR5 in solid tumors has also not been reported to improve outcome. The CCR2/CCR5 antagonist BMS-81316 is currently being tested in the neoadjuvant setting in combination with nivolumab and/or GVAX in locally advanced pancreatic ductal adenocarcinomas (NCT03767582), or with anti-IL-8 in non-small cell lung cancer (NSCLC) or hepatocellular carcinoma (HCC) (NCT04123379). Testing of this approach in patients with CNS tumors has not been reported. We posit that these approaches block both deleterious pro-tumoral and beneficial anti-tumoral subsets. As discussed above, the last years have witnessed a flurry of studies designed to map the myeloid landscape of GB at single cell resolution and in space and have unraveled the diversity of GAM phenotypes and functions. For instance, not all GAMs with an ‘inflammatory’ phenotype provide protective anti-tumoral responses. Notably, GAMs with active inflammasome-elicited IL-1β signaling contribute to tumorigenesis in preclinical models (107). This is supported by incidental results from the CANTOS phase III trial (NCT01327846) testing the anti-IL-1β antibody canakinumab on cardiovascular risk reduction in >10,000 patients with a history of myocardial infarction, that showed a marked decrease in lung cancer incidence and associated mortality (108). These findings further illustrate the potential of inflammation preventive approaches in cancer management. Efforts in mapping the GAM landscape are uncovering potential immune and metabolic therapeutic entry points that can be tested in future clinical trials in GB. Potential therapeutic targets are “myeloid checkpoints” i.e., receptors demonstrated in preclinical studies to drive the pro-tumoral and immunosuppressive activity of GAMs. These include SIRPα, TREM2, TREM1, Leukocyte immunoglobulin-like receptor subfamily B proteins (LILRB)1/2/4, Sialic acid-binding immunoglobulin-type lectin (SIGLEC)7/9/10, scavenger receptors such as Macrophage mannose receptor 1 (MRC1), also known as CD206), Macrophage receptor with collagenous structure (MARCO), Stabilin 1, also termed Clever 1, and P-selectin glycoprotein ligand-1 (PSGL-1) to name a few [reviewed in (109)] (**Figure 3**). Testing rational myeloid targeting in different settings, particularly in combination with other immunotherapies and in the neoadjuvant setting, is hoped to provide a better clinical output for patients with GB.
